# Some Rare Indo-Pacific Coral Species Are Probable Hybrids

**DOI:** 10.1371/journal.pone.0003240

**Published:** 2008-09-24

**Authors:** Zoe T. Richards, Madeleine J. H. van Oppen, Carden C. Wallace, Bette L. Willis, David J. Miller

**Affiliations:** 1 ARC Centre of Excellence for Coral Reef Studies, James Cook University, Townsville, Queensland, Australia; 2 Museum of Tropical Queensland, Townsville, Queensland, Australia; 3 Australian Institute of Marine Science, Townsville MC, Queensland, Australia; 4 School of Marine and Tropical Biology, James Cook University, Townsville, Queensland, Australia; 5 Comparative Genomics Centre, James Cook University, Townsville, Queensland, Australia; Fred Hutchinson Cancer Research Center, United States of America

## Abstract

**Background:**

Coral reefs worldwide face a variety of threats and many coral species are increasingly endangered. It is often assumed that rare coral species face higher risks of extinction because they have very small effective population sizes, a predicted consequence of which is decreased genetic diversity and adaptive potential.

**Methodology/Principal Findings:**

Here we show that some Indo-Pacific members of the coral genus *Acropora* have very small global population sizes and are likely to be unidirectional hybrids. Whether this reflects hybrid origins or secondary hybridization following speciation is unclear.

**Conclusions/Significance:**

The interspecific gene flow demonstrated here implies increased genetic diversity and adaptive potential in these coral species. Rare *Acropora* species may therefore be less vulnerable to extinction than has often been assumed because of their propensity for hybridization and introgression, which may increase their adaptive potential.

## Introduction

Corals of the genus *Acropora* are the dominant reef-builders throughout the Indo-Pacific and, although hybridization is thought to have been an important factor in their evolutionary success [1], there are few unambiguous examples of hybrids or hybrid species. In the Caribbean, where only three extant *Acropora* species are known, *A. prolifera* is the product of hybridization between the other two *Acropora* spp. [2], [3]. The low species complexity of the Caribbean coral fauna greatly simplifies unraveling such relationships. By contrast, the extraordinary species-richness of the Indo-Pacific, where over 60 *Acropora* species may occur in sympatry [Wallace & Muir, unpublished], greatly complicates the identification of hybrids.

Allele sharing between species provides evidence for introgressive hybridization [4], [5], but the unknown age of many extant Indo-Pacific species [6] makes it often difficult to distinguish between hybridization and incomplete lineage sorting (i.e. shared ancestral polymorphism) [5], [7]. For the common species on which most work to date has focused, effective (*N_e_*) and census population sizes (N) and coalescence times are unknown but potentially large and long, respectively, therefore incomplete lineage sorting cannot be ruled out. Rare species can provide new insights into the evolution of reef corals due to their intrinsically limited population sizes and therefore very short coalescence times.

*Acropora* species typically occupy reef flat, reef crest and upper reef slope habitats (i.e. 2–30 m), however, some rare species occur outside this range, and this suggests an intriguing possibility-that some rare corals may be hybrids that can occupy atypical or non-parental niches, as is the case for the Caribbean hybrid species *A. prolifera* [3]. To address to address the question of whether rare Indo-pacific *Acropora* species might also be hybrids, we analyzed DNA sequence data from nuclear and mitochondrial loci in a range of rare and common *Acropora* species from the Indo-Pacific and Caribbean.

## Materials and Methods

### Sample collection and census data

Samples (n = 1–3 individuals per species) of 14 rare and 8 common Indo-Pacific species of *Acropora* (Table 1) were collected from the Great Barrier Reef (Palm Island Group), the Marshall Islands (Rongelap Atoll) and Papua New Guinea (Kimbe Bay). Skeletal and matching tissue samples were collected from all corals sampled (n = 102 corals). Each sample was initially identified by Richards and confirmed by Wallace. All samples used for molecular analyses have matching voucher specimens registered in the World Wide *Acropora* Collection at the Museum of Tropical Queensland (www.mtq.qld.gov.au). Voucher specimens are available for inspection on request from the museum. For the purpose of this paper, rare species are those which have been recorded at <2.5% of sites for which data are available in the World Wide *Acropora* Database (which contains >20,000 records for >1,500 sites). Mean (±SE) global census sizes were estimated by >multiplying the mean global reef area available to each species by its mean local abundance per unit area (Supplementary Table S1). Mean global reef area was calculated as the sum of the mean regional reef habitat available for all regions in which each species is known to occur (Supplementary Table S2,). The proportion of reefs and sites occupied by rare species was estimated to be 10–30% of total reef area available. For present purposes, the effective population sizes were assumed to be approximately 11% of the calculated mean global census sizes (Supplementary Methods S1); this relationship is based on a comprehensive meta-analysis of data for 102 species of animals [8].

**Table 1:** Biological characteristics of species included in this study.

Species	Distribution	Range	Ecological niche	Collection location or source
***A. walindii***	Restricted	PNG	deep sandy reef slopes	Kimbe Bay, PNG
***A. rongelapensis***	Restricted	Micronesia/Indonesia	deep protected sandy slopes	Rongelap Atoll, RMI
***A. loisetteae***	Restricted	Malaysia, W. Aust, Micronesia	protected sandy lagoons	Rongelap Atoll, RMI
***A. pichoni***	Restricted	PNG, Micronesia	deep submerged shelf reefs, shipwrecks	Kimbe Bay, PNG
***A. lokani***	Restricted	SE Asia	shallow reef flat	Kimbe Bay, PNG
***A. derawanensis***	Restricted	SE Asia	protected deep sandy slopes	Kimbe Bay, PNG
***A. tenella***	Restricted	SE Asia	subtidal protected slopes, shelfs	Kimbe Bay, PNG
***A. batunai***	Restricted	Indonesia, PNG	submerged reefs, slopes	Kimbe Bay, PNG
***A. chesterfieldensis***	Restricted	Chesterfield Is., Micronesia	submerged shallow reefs	Rongelap Atoll, RMI
***A. kimbeensis***	Restricted	PNG, Micronesia	submerged reef flat	Kimbe Bay, PNG
***A. spathulata***	Restricted	GBR, PNG	reef flat and slope	Orpheus Island, GBR
***A. kirstyae***	Restricted	Indonesia, GBR, PNG, New Caledonia	protected interrefal locations	Orpheus Island, GBR
***A. papillare***	Restricted	W. Australia, GBR, Japan	ultra shallow and exposed reef	Orpheus Island, GBR
***A. speciosa***	Restricted	SE Asia, GBR, Central Pacific	subtidal, protected slopes and walls	Rongelap Atoll, RMI
***A. jacquelineae***	Restricted	Indonesia, PNG	reef slopes and submerged reefs	Kimbe Bay, PNG
***A. caroliniana***	Restricted	SE Asia-Pacific	submerged habitats	Kimbe Bay, PNG
***A. tortuosa***	Restricted	Central Pacific	subtidal, protected sandy lagoons	Rongelap Atoll, RMI
***A. granulosa***	Widespread	Indo-Pacific	reef slopes and walls	Rongelap Atoll, RMI
***A. vaughani***	Widespread	Indo-Pacific	protected subtidal habitats	Orpheus Island, GBR
***A. pulchra***	Widespread	Indo-Pacific	intertidal or shallow subtidal	van Oppen et al. 2001
***A. aspera***	Widespread	Indo-Pacific	intertidal or shallow subtidal	van Oppen et al. 2001
***A. longicyathus***	Widespread	SE Asia-Pacific	subtidal habitats	van Oppen et al. 2001
***A. loripes***	Widespread	Indo-Pacific	subtidal shallow reef habitats	Rongelap Atoll, RMI
***A. gemmifera***	Widespread	Indo-Pacific	intertidal or shallow subtidal	van Oppen et al. 2001
***A. microphthalma***	Widespread	Indo-Pacific	subtidal habitats	Orpheus Island, GBR
***A. millepora***	Widespread	Indo-Pacific	intertidal or shallow subtidal	van Oppen et al. 2001
***A. digitifera***	Widespread	Indo-Pacific	intertidal or shallow subtidal	van Oppen et al. 2001
***A. humilis***	Widespread	Indo-Pacific	intertidal or shallow subtidal	van Oppen et al. 2001
***A. austera***	Widespread	Indo-Pacific	shallow subtidal habitats	van Oppen et al. 2001
***A. cerealis***	Widespread	Indo-Pacific	shallow subtidal habitats	van Oppen et al. 2001
***A. nasuta***	Widespread	Indo-Pacific	shallow subtidal habitats	van Oppen et al. 2001
***A. valida***	Widespread	Indo-Pacific	shallow subtidal habitats	Magnetic Island, GBR
***A. palmata***	Outgroup	Atlantic Ocean	subtidal habitats	van Oppen et al. 2000
***A. prolifera***	Outgroup	Atlantic Ocean	subtial habitats	van Oppen et al. 2000
***A. cervicornis***	Outgroup	Atlantic Ocean	subtidal habitats	van Oppen et al. 2000
***I. cuneata***	Outgroup	Indo-Pacific	subtidal habitats	van Oppen et al. 2001

### DNA Extraction, Polymerase Chain Reaction, cloning and sequencing

DNA was extracted from ∼1 cm branch fragments of individual corals as previously described [5]. Markers studied were the highly polymorphic single-copy nuclear *Pax-C* 46/47 intron and the mitochondrial DNA (mtDNA) control region, for which a reference body of data exists from various *Acropora* species [5], [9]. Details of primers and procedures for PCR, cloning and sequencing are described in [5], [9]. New sequences obtained have been lodged in GenBank under EU918202-EU918288 (mitochondrial data) and EU918771-EU918925 (nuclear intron data).

### Phylogenetic Analysis

Sequences were manually aligned in Sequencher 4.5 against a subset of the existing *Acropora Pax-C* intron and mitochondrial control region sequences [2], [5], [9] before phylogenetic analysis in a Bayesian statistical framework in Mr Bayes 3.1.2 [10]. The dataset analysed therefore consisted of sequences from 17 rare and 15 common Indo-Pacific species *Acropora* species, the three Caribbean *Acropora* species and *Isopora cuneata*. Genetic distances were calculated as Kimura 2-parameter distances [11]. The optimal model of sequence evolution was identified using hierarchical likelihood ratio tests in MrModeltest 2.2 [12]. The (MCMC) analyses were run for 5 million generations, with burn-in times of 20,000–50,000 (p<0.05). Trees generated from the *Pax-C* data were rooted using sequences from *Isopora cuneata,* whereas the mtDNA tree was rooted with *A. cervicornis* as in this case the degree of divergence of the *I. cuneata* sequence effectively precluded unambiguous alignment. Analyses were conducted on the full alignments as the exclusion or weighting down of large indels or repeat regions was found not to significantly effect the overall topology (see also [5]).

## Results

**Figure 1:**
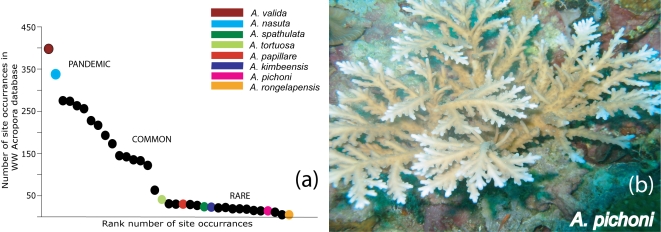
(a). Global abundance of the *Acropora* species used in this study. These data are based on numbers of records in the World Wide *Acropora* Database (n = 1523 sites; [6] and Wallace unpublished). (b). Several rare species, such as *A. pichoni* shown here, are likely to be unidirectional hybrids and occupy atypical habitats. Photo credit: Maria Beger.

Allele/haplotype data from nuclear and mitochondrial loci were determined for 17 rare and 15 common Indo-Pacific *Acropora* species as well as all 3 Caribbean species of *Acropora* (Table 1) and *Isopora cuneata*. Only samples from taxonomically unambiguous individuals were included in this study; the morphology of the corals sampled was absolutely consistent with their formal description [6]. To avoid the possibility of sampling clonemates, corals sampled were separated by at least 10 meters. The extreme rarity of several of the species examined limited the number of samples that it was possible to examine. Plots of the number of species distribution records against rank order (Figure 1a) clearly resolve rare species, such as *A. pichoni* (Figure 1b), from common species, with *A. valida* and *A. nasuta* being essentially pandemic throughout the Indo-Pacific.

### Census Sizes

**Figure 2:**
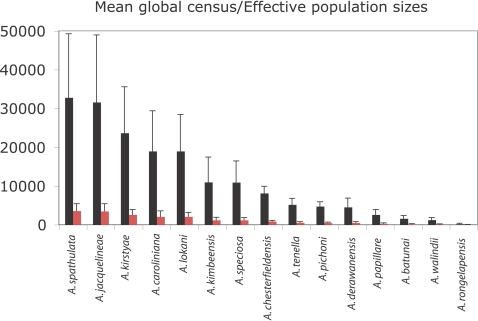
Effective population size data for rare *Acropora* species included in this study. Mean (±SE) global census sizes are shown as black histograms, and predicted effective population sizes as red histograms. Data for *A. tortuosa* are omitted, as the mean global census size for this species (Supplementary Table S1) is more than two-fold higher than for *A. spathulata* (of those shown, the species with the largest global census size).

Effective population sizes in reef corals are expected to be significantly smaller than census sizes for a number of reasons [13]. First, corals are known to undergo extreme variation in census population sizes due to perturbations such as storms and cyclones, bleaching or crown-of-thorns starfish outbreaks, which will substantially reduce effective sizes because it diminishes the proportion of the population involved in reproduction [8]. Second, high variance in fecundity (which is again known in corals [14]) reduces *N*_e_ because neither juveniles nor senescent adults take part in reproduction [15]. Third, some *Acropora* species reproduce asexually by fragmentation or fission [16], which again reduces *N*_e_. Here we find mean (±SE) global census population sizes for rare species in this study varied from 32823 (±16412) for *A. spathulata* to 224 (±117) for *A. rongelapensis*. Based on the *N_e_* estimate of 11% of the census population size, *A. spathulata* has a mean effective global population size of 3611 (±1805) and A. *rongelapensis*, 25 (±13) (Figure 2). Furthermore, it is likely that local population census and effective population sizes are substantially smaller than these conservative global estimates.

### Pax-C intron data

**Figure 3:**
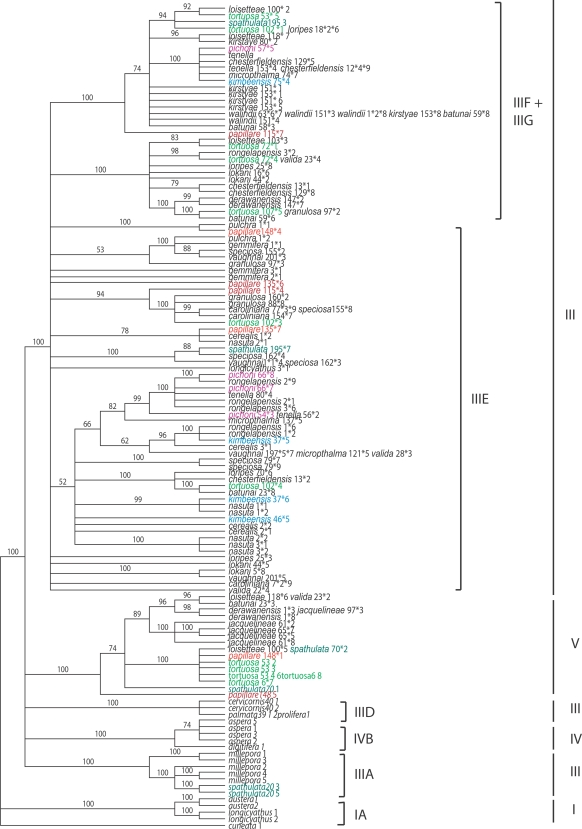
Phylogenetic analysis of PaxC data. The figure shows the majority rule (>50%) consensus tree obtained in a Bayesian analysis of nuclear sequence data for the thirty-five *Acropora* species included in this study, with *Isopora cuneata* defined as outgroup. Bayesian analyses used likelihood settings from best-fit model (HKY+G) selected by hLRT in MrModeltest 2.2 [12]: 5 million generations; burn in  = 50,000. Numbers above branches are posterior probability values supporting the topology shown and clades are labelled according to previous [5], [9] analyses. Numbers after species names indicate the coral colonies from which the sequences were obtained; where more than one sequence was obtained per colony, the clone identity is given after an asterisk. Note that in some cases multiple clones (sometimes from different species) had identical sequences.

Results of phylogenetic analyses of Pax-C intron data (Figure 3) are broadly consistent with previous results, but some details differ due to the selection of taxa. To facilitate comparison with previous analyses, clades are labeled according to published trees [5], [9]. As in previous analyses, the basal clade contains *A. longicyathus*, and, in the present case, *A. austera*. In the present tree, a polytomy then gives rise to strongly supported clades corresponding to IIIA, IVB, IIID of previous studies; a major difference is the novel clade V which is composed exclusively of rare species with the exception of a single allele from *A. valida*. The nuclear tree distinguishes the Caribbean species in the highly supported clade IIID. Within the large terminal clade, two novel subclades (III F+G) were identified, containing predominantly sequences from rare species.

### Mitochondrial control region data

**Figure 4:**
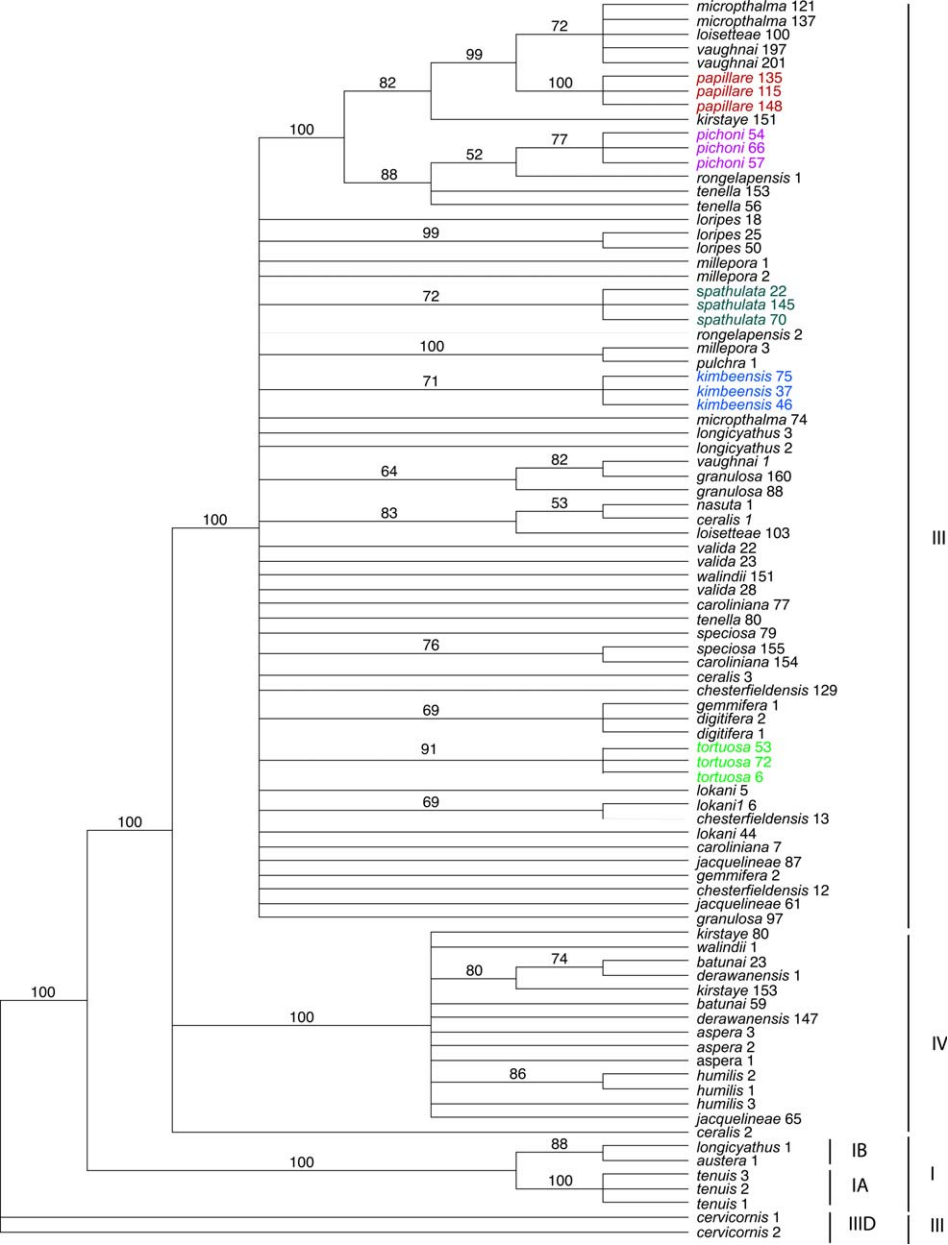
Phylogenetic analysis of mitochondrial sequence data. The figure shows the majority rule (>50%) consensus tree obtained in a Bayesian analysis of mitochondrial sequence data for thirty-five Indo-Pacific *Acropora* species with the Caribbean species *Acropora cervicornis* defined as outgroup. Bayesian analysis used likelihood settings from best-fit model (HKY+I+G) selected by hLRT in MrModeltest 2.2 [12]: 5 million generations; burn in  = 20,000. Numbers above branches are posterior probability values supporting the topology shown and clades are labelled according to previous [5], [9] analyses. Numbers after species names indicate the coral colonies from which the sequences were obtained.

Phylogenetic analyses of the mtDNA Control Region (Figure 4) were also broadly consistent with previous results and clades were labeled as in previous publications [5], [9]. The basal clade (IA/IB) again contains *A. longicyathus* and *A. austera*, with *A. tenuis* added. In the present case, clade III is expanded and clade IV contracted relative to published analyses, due to differences in composition of the datasets. Clade IV includes *A. aspera, A. humilis* and several rare species (e.g. *A. kirstyae, A. derawanensis*).

## Discussion

In both the Pax-C and mitochondrial phylogenies many *Acropora* species are polyphyletic. Previous work [5], [9] provides precedents for this pattern, which has been interpreted as evidence for interspecific hybridization. However, the Indo-Pacific species examined in these previous studies are widespread and locally common, and in these cases lineage sorting will occur slowly. As the fossil record of *Acropora* is extremely limited, for common and widespread species incomplete lineage sorting cannot be rigorously excluded as an alternative explanation for the observed polyphyletic patterns. However, for the rare species included in the present study, effective population sizes are so small (Fig 2) that lineage sorting will occur on very short time scales, so in contrast to the position with common species, polyphyletic patterns observed for rare species provide unequivocal evidence for hybridization.

**Figure 5:**
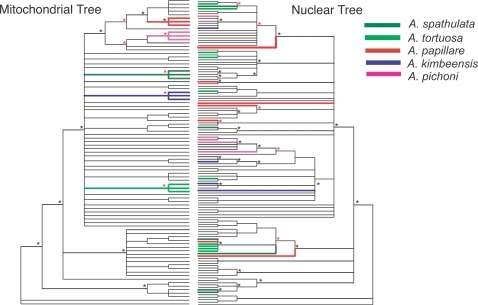
Comparison of nuclear and mitochondrial phylogenies. Asterisks indicate posterior probability values of 100% (black) or >70% (red); for clarity, asterisks are shown only at nodes affecting the positions of sequences from *A. papillare*, *A. pichoni*, *A. kimbeensis*, *A. spathulata* and *A. tortuosa.*

Comparison of the trees generated from nuclear and mitochondrial data (Figure 5) shows that three of the rare species studied here-*A. pichoni*, *A. kimbeensis* and *A. papillare*-are monophyletic for the mtDNA marker but are polyphyletic and contain highly divergent alleles at the nuclear marker, even within individual corals. The presence of species-specific mitochondrial haplotypes is unusual in *Acropora* [5], [9]. Of the 49 species studied to date, the only other *Acropora* species that is monophyletic in mtDNA is *A. tenuis* (Figure 4; however, see also below), which is known to be reproductively isolated through a difference in spawning time [5].

The mitochondrial phylogeny implies that the three monophyletic rare species have evolved relatively recently, because they fall within derived positions of the large terminal clade that reflects the post-Miocene Indo-Pacific speciation of *Acropora* (i.e. <5.32 my) [5], [17]. In contrast, sequences from these three species are widely distributed throughout the nuclear tree; for example, alleles from *A. papillare* occur in both Clades III and V. This pattern in nuclear versus mtDNA loci can be explained by the known faster lineage sorting of mitochondrial haplotypes than alleles at single copy nuclear loci [18]. Unlike their more common relatives, the small effective global population sizes of these three rare species (*A. pichoni* = 521±125; *A. kimbeensis* = 1208±707; *A. papillare* = 284±142) effectively rules out the possibility of incomplete lineage sorting, because of their small population sizes, these rare species have very short coalescence times.

There is no evidence that these rare species were historically more common. Moreover, these observed patterns–monophyly with respect to mitochondrial haplotypes accompanied by polyphyly at nuclear loci-cannot be explained as consequences of either recent population crashes or population bottlenecks. Under a population crash scenario one would expect to find divergent mitochondrial haplotypes as well as divergent nuclear alleles, whereas under a population bottleneck scenario (i.e. a crash occurring less recently) low diversity at both nuclear and mitochondrial loci is expected. These alternate possibilities can therefore be ruled out, and the most parsimonious explanation for the observed patterns of allele/haplotype distribution is that *A. pichoni*, *A. kimbeensis* and *A. papillare* are unidirectional hybrids.

In the Caribbean, the hybrid species *A. prolifera* colonizes habitats that are distinct from those of the parental species [2], [3]. Similarly, two of the three rare putative hybrid species from the Indo-Pacific, *A. pichoni* and *A. papillare,* occur in atypical habitats. Whereas the vast majority of *Acropora* spp. occur in relatively shallow reef flat, crest and slope habitats (2–30 m), *A. pichoni* occurs below 40 m and *A papillare*, is found in extremely shallow intertidal habitats (<2 m). Specialization in extremely shallow or deep habitats is atypical for *Acropora* species hence our data provide support for the hypothesis that hybrid species may exploit atypical (or non-parental) niches.

Other rare species occurring in small and isolated populations (e.g. *A. walindii, A. loisetteae, A. derawanensis* and *A. jacquelineae*) are polyphyletic with respect to both nuclear alleles and mitochrondrial haplotypes. Whilst these patterns are again consistent with hybridization, in these cases alternative explanations, such as recent population crashes, cannot be rigorously excluded.

Two species that are geographically restricted but locally common (*A. spathulata* and *A. tortuosa)* are also monophyletic at the mitochondrial marker but polyphyletic at the nuclear marker. However, in these latter cases, incomplete lineage sorting cannot be ruled out because of the longer coalescence times for these species resulting from their larger census and predicted effective population sizes.

The results presented here imply that a number of rare Indo-Pacific *Acropora* species are the products of recent hybridization events, and highlight the significance of hybridization in coral diversification. Whether these species have hybrid origins or have evolved and then hybridized in the absence of conspecific gametes remains to be elucidated.

In summary, although it has often been assumed that small populations have a decreased potential for adaptation [19], our analyses imply that some rare Acroporid corals may actually have increased adaptive potential as a consequence of introgressive hybridization [20], and therefore may be less vulnerable to extinction than has been assumed.

## Supporting Information

Methods S1 Calculation of mean global census and effective population sizes
(0.07 MB DOC)

Table S1 Estimates of mean global census size for rare species included in this study.
(0.12 MB DOC)

Table S2 Regional estimates of available reef habitat post 2004.
(0.09 MB DOC)
