# Correction: Some Rare Indo-Pacific Coral Species Are Probable Hybrids

**DOI:** 10.1371/journal.pone.0211527

**Published:** 2019-01-25

**Authors:** Zoe T. Richards, Madeleine J. H. van Oppen, Carden C. Wallace, Bette L. Willis, David J. Miller

The equation used to estimate population sizes for this article [1] is incorrect. The error occurred when standardizing the mean area available to target species in the equation which was listed as 1000 (km^2^) in the supplementary methods but should have read 1000^2^ (m). This means that the equation for estimating mean global population sizes has an error and needs to be modified (see below for updated supplementary S1 Methods). As a result, the mean global census sizes as listed in S1 Table are 1000 times smaller than they should be. The revised supplementary S1 Table is included below with the revisions in red.

The implication of this error is that the estimated mean global census sizes should be 1000 times larger than currently reported; however, this does not change the interpretation of what is rare and what is common. The error was applied across the board to all species in the analysis. Hence, the rare species still have smaller n^global^ sizes than the common species and as such, their coalescence times are still shorter in comparison to the more common species.

This three-order of magnitude change in global population sizes does, however, affect the wording in the article: statements referring to abundance should be made in relative terms, and time scales on which lineage sorting occurs in different species should be stated in relative rather than absolute terms. Thus, we provide revisions to the following sentences:

The fourth sentence of the first paragraph of the Results section is revised to: The relative rarity of several of the species examined limited the number of samples that it was possible to examine.

There are errors in the fifth and sixth sentences of the Census Sizes subsection of the Results. The correct sentences are: Here we find mean (**±** SE) global census population sizes for rare species in this study varied from (32823 **±** 16412) ×10^3^ for *A*. *spathulata* to (224 ± 117) ×10^3^ for *A*. *rongelapensis*. Based on the Ne estimate of 11% of the census population size, *A*. *spathulata* has a mean effective global population size of (3611 ± 1805) ×10^3^ and *A*. *rongelapensis*, (25 ± 13) ×10^3^ (Fig 2).

**Fig 2:**
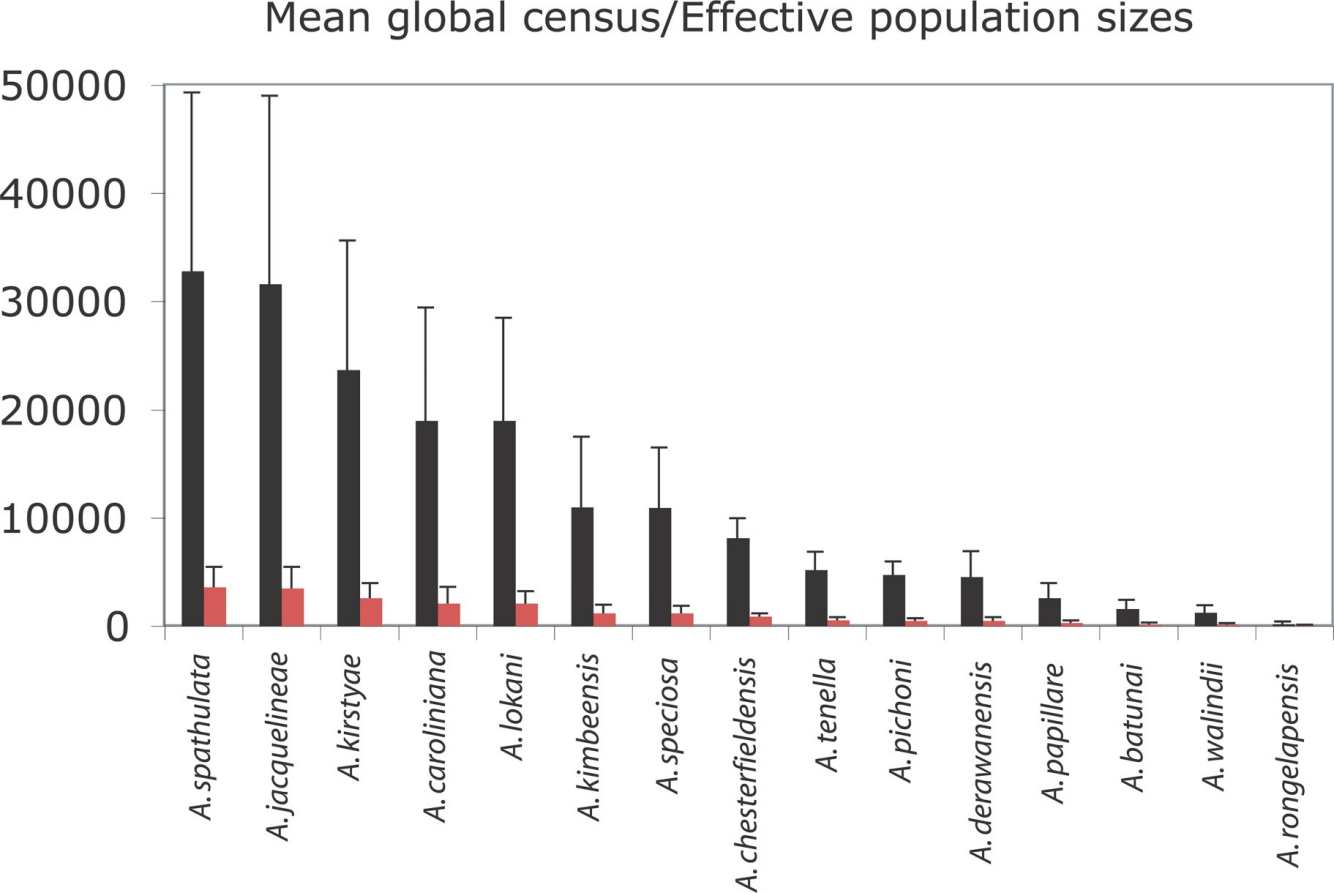
Effective population size data for rare *Acropora* species included in this study. Mean (**±** SE) (x10^3^) global census sizes are shown as black histograms, and predicted effective population sizes as red histograms. Data for *A*. *tortuosa* are omitted, as the mean global census size for this species (S1 Table) is more than two-fold higher than for *A*. *spathulata* (of those shown, the species with the largest global census size).

There are errors in the fourth sentence of the third paragraph in the Discussion section. The correct sentence is: Unlike their more common relatives, the small effective global population sizes of these three relatively rare species {*A*. *pichoni* = (521 ± 125) ×10^3^; *A*. *kimbeensis* = (1208 **±** 707) ×10^3^; *A*. *papillare* = (284 **±** 142) ×10^3^)} effectively rules out the possibility of incomplete lineage sorting, because of their comparatively small population sizes, these relatively rare species have shorter coalescence times.

There are errors in the caption for Fig 2. Please see the complete, correct Fig 2 caption here.

## Supporting information

S1 Methods Calculation of mean global census and effective population sizes. (DOCX)

S1 Table Estimates of mean global census size for rare species included in this study. (DOCX)
