# Supplementary material for: Some Rare Indo-Pacific Coral Species Are Probable Hybrids
Source: PLoS One. 2008 Sep 24;3(9):e3240. doi: 10.1371/journal.pone.0003240 (PMC2531234; doi:10.1371/journal.pone.0003240)
Supplement: Table S1 — Estimates of mean global census size for rare species included in this study. (0.12 MB DOC) [file pone.0003240.s002.doc]

| **Species** | **Region** | **Combined Global Reef Area (km^2^)** | **Habitat** | **% site available per habitat** | **Reef area available to rare sp (km^2^)** | **Mean local ab.** | **Mean**  **Global Census size**  **(n^global^)** | **SE** | **Mean global**  **Ne +/- SE** |
| --- | --- | --- | --- | --- | --- | --- | --- | --- | --- |
| ***papillare*** | SE Asia  Australia  Sth Asia | 25812.7 +/- 12906.35 | intertidal | 10% | 2581.27 +/- 1290.64 | 3 | 2581.27 | 1290.64 | **284 +/- 142** |
| ***spathulata*** | Australia | 12308.8 +/- 6154.4 | reef flat | 40% | 4923.52 +/- 2461.76 | 20 | 32823.47 | 16411.73 | **3611+/- 1805** |
| ***batunai*** | SE Asia-PNG | 23699.6 +/- 11849.8 | deep slope | 10% | 2369.96 +/- 1184.98 | 2 | 1579.97 | 789.98 | **174 +/-87** |
| ***pichoni*** | SE Asia  PNG | 23699.6 +/- 11849.8 | deep slope | 10% | 2369.96 +/- 1184.98 | 6 | 4739.92 | 1139.08 | **521 +/- 125** |
| ***tenella*** | SE Asia  Micronesia  PNG | 26036.4 +/- 8032.2 | deep slope | 10% | 2603.64 +/- 803.22 | 6 | 5207.28 | 1606.44 | **573 +/- 177** |
| ***walindii*** | PNG | 12308.8 +/- 6154.4 | deep slope | 10% | 1230.88 +/- 615.44 | 3 | 1230.88 | 615.44 | **135 +/- 68** |
| ***tortuosa*** | Micronesia  Polynesia  SW Pacific | 20297.1 +/- 4453.05 | inter-reefal sand | 100% | 20297.1 +/- 4453.05 | 10 | 67657 | 14843.5 | **7442 +/-**  **1633** |
| ***derawanensis*** | SE Asia | 11390.8 +/- 5695.4 | shallow slope | 40% | 4556.32 +/- 2278.16 | 3 | 4556.32 | 2278.16 | **501 +/- 251** |
| ***kirstyae*** | SE Asia  Australia | 23699.6 +/- 11849.8 | inter-reefal sand | 100% | 23699.6 +/- 11849.8 | 3 | 23699.6 | 11849.8 | **2606 +/-**  **1303** |
| ***speciosa*** | SE Asia  Micronesia  PolynesiaPNG | 27356.06 +/- 13678.03 | shallow slope | 40% | 10942,44 +/- 5471.2 | 3 | 10942.44 | 5471.21 | **1204 +/-**  **601** |
| ***caroliniana*** | SE Asia  Australia | 23699.6 +/- 13018.2 | shallow slope | 40% | 9479.84 +/- 5207.28 | 6 | 18959.68 | 10414.56 | **2086 +/- 1456** |
| ***chesterfieldensis*** | Micronesia  Polynesia  SW Pacific | 20297.1 +/- 4453.05 | reef flat | 40% | 8118.8 +/-1781.22 | 3 | 8118.8 | 1781.22 | **893 +/- 196** |
| ***rongelapensis*** | Micronesia | 2336.8 +/-1168.4 | deep slope | 10% | 233.68 +/- 116.84 | 3 | 223.68 | 116.84 | **25 +/- 13** |
| ***jacquelineae*** | SE Asia  PNG | 23699.6 +/- 11849.8 | shallow slope | 40% | 9479.84 +/- 5207.28 | 10 | 31 599.47 | 17 357.6 | **3476 +/- 1909** |
| ***lokani*** | SE Asia  PNG | 23699.6 +/- 11849.8 | reef flat-shallow slope | 40% | 9479.84 +/- 5207.28 | 6 | 18 959.68 | 9479.84 | **2086 +/- 1043** |
| ***kimbeensis*** | SE Asia  Micronesia | 13727.6 +/- 6863.8 | reef flat | 40% | 5491.04 +/- 2745.52 | 6 | 10 982.08 | 6425.76 | **1208 +/- 707** |
| ***loisetteae*** | SE Asia  Australia | 23699.6 +/-11849.8 | inter-reefal sand | 100% | 23699.6 +/-11849.8 | 10 | 78998.66 | 39499.33 | **8690 +/- 4345** |
