# Supplementary material for: Some Rare Indo-Pacific Coral Species Are Probable Hybrids
Source: PLoS One. 2008 Sep 24;3(9):e3240. doi: 10.1371/journal.pone.0003240 (PMC2531234; doi:10.1371/journal.pone.0003240)
Supplement: Table S2 — Regional estimates of available reef habitat post 2004. (0.09 MB DOC) [file pone.0003240.s003.doc]

| **Region** | **Total reef area (km2)** | **Percent of reefs destroyed >2004** | **Reef remaining after 2004 (km2)** | **10-30% region occupied by rare species** | **Mean**  **available**  **regional reef habitat** | **SE** |
| --- | --- | --- | --- | --- | --- | --- |
| **S Asia** | 19210 | 45 | 10565.5 | 3169.65 | 1056.55 | 1056.55 |
| **SE Asia** | 91700 | 38 | 56854 | 17086.2 | 5695.4 | 5695.4 |
| **E & N Asia** | 5400 | 14 | 4644 | 1393.2 | 464.4 | 464.4 |
| **Australia & PNG** | 62800 | 2 | 61544 | 18463.2 | 6154.4 | 6154.4 |
| **SW Pacific** | 27060 | 3 | 26248.2 | 7874.86 | 2624.82 | 2624.82 |
| **Polynesia** | 6733 | 2 | 6598.34 | 1979.49 | 659.83 | 659.83 |
| **Micronesia** | 12700 | 8 | 11684 | 3505.2 | 1168.4 | 1168.4 |
