# Supplementary material for: Correction: Some Rare Indo-Pacific Coral Species Are Probable Hybrids
Source: PLoS One. 2019 Jan 25;14(1):e0211527. doi: 10.1371/journal.pone.0211527 (PMC6347392; doi:10.1371/journal.pone.0211527)
Supplement: S1 Methods — (DOCX) [file pone.0211527.s001.docx]

**S1 Methods. Calculation of mean global census and effective population sizes.**

Global census size estimates (N) were calculated to enable estimation of effective population sizes (Ne) for the rare species included in this study. The mean global census sizes of rare species (+SE) were estimated using global estimates of reef area to calculate mean global census size. Regional estimates of total reef area (e.g. SE Asia, Micronesia) were extracted from Wilkinson (Status of Coral Reefs of the World, Australian Institute of Marine Science, 2004), factoring in percentages of the total reef area available destroyed prior to 2004. Reef remaining after 2004 was calculated for each region based on data Supplementary Table 2. We assume that rare species occupy a small proportion of the reef available within the regions that it is present, hence we calculate that 10-30% of regional reef habitat is available to rare species by calculating the mean (+SE).

The combined global reef area available to each species was calculated from the mean available regional reef habitat km^2^ (+SE) summed for all regions each species is known to occur within. Habitat preferences were assigned to each species; this determined the percentage of a site available to target species according to their habitat preference. For example 10% of a site is available to intertidal species, 40% is available to reef flat/crest species, 40% is available to shallow slope species <30m, and 10% of a site is available to deep slope species >30m. The result is an estimate of the reef area available to target rare species. The mean local abundance was determined for each species from relative estimates of local population size (Richards unpublished). To determine the mean global census size from the reef available to target species, we estimated that one RAP biodiversity swim (Rapid Visual Assessment) covers approximately 3000 m^2^, thus to estimate mean global census size (n^global^) we standardize the distance measure by multiplying reef area available to target species (km^2^) (+SE) by 1000 m^2^ and divide by 3000 m^2^ (average area covered on a single rapid visual biodiversity assessment), and multiply by the mean local site abundance.

N ^global^ = Reef area available to rare species x 1000^2^ x mean local abundance

3000
