# Supplementary material for: Correction: Some Rare Indo-Pacific Coral Species Are Probable Hybrids
Source: PLoS One. 2019 Jan 25;14(1):e0211527. doi: 10.1371/journal.pone.0211527 (PMC6347392; doi:10.1371/journal.pone.0211527)
Supplement: S1 Table — (DOCX) [file pone.0211527.s002.docx]

**S1 Table. Estimates of mean global census size for rare species included in this study.**

| **Species** | **Region** | **Combined Global Reef Area (km^2^)** | **Habitat** | **% site available per habitat** | **Reef area available to rare sp (km^2^)** | **Mean local ab.** | **Mean**  **Global Census size**  **(n^global^)**  **x10^3^** | **SE x10^3^** | **Mean global**  **(Ne ± SE) x10^3^** |
| --- | --- | --- | --- | --- | --- | --- | --- | --- | --- |
| ***papillare*** | SE Asia  Australia  Sth Asia | 25812.7 ± 12906.35 | intertidal | 10% | 2581.27 ± 1290.64 | 3 | 2581.27 | 1290.64 | **284 ± 142** |
| ***spathulata*** | Australia | 12308.8 ± 6154.4 | reef flat | 40% | 4923.52 ± 2461.76 | 20 | 32823.47 | 16411.73 | **3611± 1805** |
| ***batunai*** | SE Asia-PNG | 23699.6 ± 11849.8 | deep slope | 10% | 2369.96 ± 1184.98 | 2 | 1579.97 | 789.98 | **174 ± 87** |
| ***pichoni*** | SE Asia  PNG | 23699.6 ± 11849.8 | deep slope | 10% | 2369.96 ± 1184.98 | 6 | 4739.92 | 1139.08 | **521 ± 125** |
| ***tenella*** | SE Asia  Micronesia  PNG | 26036.4 ± 8032.2 | deep slope | 10% | 2603.64 ± 803.22 | 6 | 5207.28 | 1606.44 | **573 ± 177** |
| ***walindii*** | PNG | 12308.8 ± 6154.4 | deep slope | 10% | 1230.88 ± 615.44 | 3 | 1230.88 | 615.44 | **135 ± 68** |
| ***tortuosa*** | Micronesia  Polynesia  SW Pacific | 20297.1 ± 4453.05 | inter-reefal sand | 100% | 20297.1 ± 4453.05 | 10 | 67657 | 14843.5 | **7442 ±**  **1633** |
| ***derawanensis*** | SE Asia | 11390.8 ± 5695.4 | shallow slope | 40% | 4556.32 ± 2278.16 | 3 | 4556.32 | 2278.16 | **501 ± 251** |
| ***kirstyae*** | SE Asia  Australia | 23699.6 ± 11849.8 | inter-reefal sand | 100% | 23699.6 ± 11849.8 | 3 | 23699.6 | 11849.8 | **2606 ±**  **1303** |
| ***speciosa*** | SE Asia  Micronesia  Polynesia PNG | 27356.06 ± 13678.03 | shallow slope | 40% | 10942,44 ± 5471.2 | 3 | 10942.44 | 5471.21 | **1204 ±**  **601** |
| ***caroliniana*** | SE Asia  Australia | 23699.6 ± 13018.2 | shallow slope | 40% | 9479.84 ± 5207.28 | 6 | 18959.68 | 10414.56 | **2086 ± 1456** |
| ***chesterfieldensis*** | Micronesia  Polynesia  SW Pacific | 20297.1 ± 4453.05 | reef flat | 40% | 8118.8 ± 1781.22 | 3 | 8118.8 | 1781.22 | **893 ± 196** |
| ***rongelapensis*** | Micronesia | 2336.8 ± 1168.4 | deep slope | 10% | 233.68 ± 116.84 | 3 | 223.68 | 116.84 | **25 ± 13** |
| ***jacquelineae*** | SE Asia  PNG | 23699.6 ± 11849.8 | shallow slope | 40% | 9479.84 ± 5207.28 | 10 | 31 599.47 | 17 357.6 | **3476 ± 1909** |
| ***lokani*** | SE Asia  PNG | 23699.6 ± 11849.8 | reef flat-shallow slope | 40% | 9479.84 ± 5207.28 | 6 | 18 959.68 | 9479.84 | **2086 ± 1043** |
| ***kimbeensis*** | SE Asia  Micronesia | 13727.6 ± 6863.8 | reef flat | 40% | 5491.04 ± 2745.52 | 6 | 10 982.08 | 6425.76 | **1208 ± 707** |
| ***loisetteae*** | SE Asia  Australia | 23699.6 ± 11849.8 | inter-reefal sand | 100% | 23699.6 ± 11849.8 | 10 | 78998.66 | 39499.33 | **8690 ± 4345** |
